# Mating dynamics in a nematode with three sexes and its evolutionary implications

**DOI:** 10.1038/srep17676

**Published:** 2015-12-03

**Authors:** Jyotiska Chaudhuri, Neelanjan Bose, Sophie Tandonnet, Sally Adams, Giusy Zuco, Vikas Kache, Manish Parihar, Stephan H. von Reuss, Frank C. Schroeder, Andre Pires-daSilva

**Affiliations:** 1 University of Texas at Arlington, Department of Biology, Arlington, USA; 2 Boyce Thompson Institute and Department of Chemistry and Chemical Biology, Cornell University, Ithaca, New York 14853, USA; 3 University of Warwick, School of Life Sciences, Coventry, United Kingdom

## Abstract

Nematodes have diverse reproductive strategies, which make them ideal subjects for comparative studies to address how mating systems evolve. Here we present the sex ratios and mating dynamics of the free-living nematode *Rhabditis* sp. SB347, in which males, females and hermaphrodites co-exist. The three sexes are produced by both selfing and outcrossing, and females tend to appear early in a mother’s progeny. Males prefer mating with females over hermaphrodites, which our results suggest is related to the female-specific production of the sex pheromones ascr#1 and ascr#9. We discuss the parallels between this system and that of parasitic nematodes that exhibit alternation between uniparental and biparental reproduction.

Organisms have evolved several modes of reproduction, varying from species with separate sexes to species that replicate exclusively from a single parent^1^,^2^. Between these extremes lie mixed breeding systems, including gynodioecy (females and hermaphrodites), androdioecy (males and hermaphrodites), and trioecy (males, females and hermaphrodites). The co-existence of self-fertilizing (selfing) hermaphrodites with individuals that reproduce mainly by outcrossing has long puzzled evolutionary biologists since Darwin^3^, as mixed breeding systems are predicted to be rare and evolutionarily unstable^4^,^5^.

The majority of studies on mixed breeding systems have focused on flowering plants, where hermaphroditism has evolved into species with separate sexes on multiple occasions^6^,^7^. However, comparatively little is known about these transitions in animals, for which most seem to have occurred in the opposite direction, from separate sexes to hermaphroditism^1^,^8^,^9^.

Theoretical and empirical studies support a model in which an ancestral gonochoristic (with separate sexes) population was invaded by a mutant selfing hermaphrodite, creating a trioecious population^10^,^11^,^12^,^13^,^14^. Theory predicts that trioecy is a transient state^15^. Consistent with this theory, this breeding system is relatively rare in animal taxa^1^,^16^,^17^. Nematodes exhibit a wide diversity of breeding systems, including trioecy^18^,^19^,^20^,^21^,^22^,^23^,^24^,^25^. Thus, this animal taxon is an interesting group to study how trioecy evolves and is maintained. However, the majority of the trioecious nematode species are parasitic and thus quite challenging to study^26^,^27^,^28^,^29^,^30^.

In this study, we focus on the nematode *Rhabditis* sp. SB347, a unique free-living trioecious species that is far more amenable to laboratory manipulation^31^,^32^. Although not yet formally described, phylogenetic analysis using molecular markers places SB347 in Eurhabditids^33^, a species-rich clade above the genus level that includes the model nematode *Caenorhabditis elegans*^29^. At present very little is known about the ecology of SB347, which was originally isolated from a deer tick used as nematode bait^29^.

The male versus non-male sex is chromosomally determined^34^. Males are relatively rare in SB347, comprising about 13% of self-progeny of hermaphrodites^31^ and 1.6–2.3% of the outcrossed progeny of females^31^,^34^.

SB347 adult hermaphrodites and females are identical in karyotype and adult somatic phenotype, but the germline of the former produces sperm and oocytes^31^. The mechanism by which a selfing hermaphrodite produces progeny of all three sexes is not currently clear. However, a previous study suggests that the age of the selfing parent is an important factor^32^, because an older mother tends to produce a higher percentage of hermaphrodite progeny than a younger mother (Fig. 1).

An important developmental difference between SB347 hermaphrodites and females occurs during the larval stages. Before becoming adults, nematodes typically undergo four larval molts named L1–L4 (Fig. 1). In *C. elegans* and other free living nematodes, unfavorable growth conditions encountered by the L1 larvae determine the formation of the stress-resistant larval morph named ‘dauer’. However, in SB347 a significant proportion of larvae seem to be already pre-specified to become dauers, independently of the environmental conditions experienced by the L1s. Those L1 larvae of SB347 pre-specified to become dauers have a much smaller gonad primordium when compared to siblings of the same age that do not undergo dauer formation^31^,^32^ (Fig. 1). If conditions remain favorable for growth, those larvae exit dauer within 24 h and develop into hermaphrodite adults, but never into females or males^31^,^32^. Thus, SB347 dauer formation and hermaphroditism are developmentally linked^32^. In fact, by manipulating hormones that regulate dauer formation, it is possible to convert larvae pre-specified as hermaphrodite to develop into female adults and vice-versa^32^.

To gain insights into why hermaphrodites do not entirely replace females in SB347, the aim of this study was to characterize in more detail how each sex is generated and maintained in this trioecious species. We found that female and hermaphrodite progeny are produced at different points in the reproductive life of mothers, probably indicating some ecological distinction between hermaphrodites and females. Furthermore, we characterized the mating behavior in this trioecious system. Our results indicate that outcrossing is more likely to occur between males and females. The results are discussed in the light of the evolution of life cycles with alternating modes of reproduction, such as those found in some parasitic nematodes.

## Methods and Materials

### Nematode culture

*Rhabditis* sp. SB347 was fed with the streptomycin-resistant *Escherichia coli* OP50-1 strain and cultured following standard conditions at 20 °C, as used for *C. elegans*^35^. Microbial contamination was prevented by using 200 μg/mL nystatin and 200 μg/mL streptomycin in the nematode growth medium (NGM)^36^. The SB347 strain used in this study, originally provided by Karin Kiontke (New York University), was inbred for 50 generations by selfing a single hermaphrodite in every generation. The resultant inbred strain was named APS4. Although we used this inbred strain for all experiments in the present study, we herein referred to it simply as SB347.

### Sexing progeny of crossing and selfing parents

**Figure 2:**
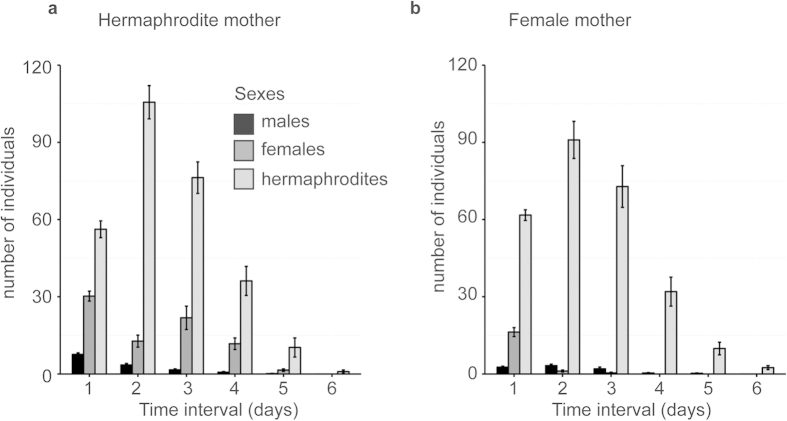
Sexes of F1 from selfing or outcrossing parents. Total number of males, females and hermaphrodites produced by (**a**) hermaphrodites and (**b**) females each day of their reproductive life. Error bars are SEM.

To determine the sex of the F1 from selfing hermaphrodites, the self-progeny of 20 SB347 animals was collected throughout their lifespan (Fig. 2a). The hermaphrodite mothers were selected by first isolating dauers, which in SB347 always develop into hermaphrodites^32^. Dauers were placed into single 6 cm agar plates seeded with *E. coli* OP50-1 and left to mature into adults. Every 8 h of their reproductive life, the hermaphrodite mothers were transferred to a new plate until they stopped laying eggs. To control more precisely for worms that may escape the plate before being scored, we sexed the progeny while they were at larval stages (two days after hatching). We scored the three sexes using several traits, including developmental rates and morphologies. Dauer larvae (destined to become hermaphrodites) are thin with darkly-pigmented guts. Larger larvae with long thin tails develop into females. Male larvae have a characteristically blunt tail.

The variation in F1 female/hermaphrodite frequencies in relation with the age of the mother was modeled using a Generalized Linear Model (GLM). The counts of female and hermaphrodite progeny (binomial error distribution and logit-link function) were used as the response variable. The age of the mother, which was measured according to the first day she laid an egg, was set as the explanatory variable. The model was built using the *glm* function in the ‘stats’ package in R. The statistical significance of the “day” effect on the female/hermaphrodite proportion was assessed by the Wald *chi*-squared test using the ANOVA function (type II) from the ‘car’ package^37^.

To determine the sex ratios of the progeny of females, 20 crosses were performed (Fig. 2b). Each crossing experiment consisted of one male and one female placed for a few hours (1–5 h) in an agar plate spotted with a ~5 mm diameter *E. coli* OP50-1 lawn. To ensure fertilization over the entire lifespan of the female, the female was mated with a new male every day. On the first day, one of the mothers escaped from the plate but the data of the sex of its progeny were nevertheless considered for the 8 h interval she was present. The sexes of the progeny were determined as described above. Crosses typically resulted in high percentage of XX progeny due to the preferential production of viable X-bearing sperm by the XO father^34^.

We could not determine the sex ratios of hermaphrodite adults crossed with males because it is not possible to distinguish the self- from the cross-progeny. We attempted to use old hermaphrodite parents, which by the 4^th^–5^th^ day of adulthood have depleted their limited supply of self-sperm. However, those worms died shortly after mating and did not produce progeny.

### Sperm size

To release the sperm and measure their size, ten animals of each sex were cut using a 22 gauge needle in the presence of 5–10 μL drop of sperm buffer (90 mM NaCl, 50 mM KCl, 2 mM MgCl_2_, 10 mM CaCl_2_, 100 mM HEPES, and 20 mM dextrose to pH 7.8 for a 2× solution)^34^. Males were cut at the level of the *vas deferens* and hermaphrodites at the level of the spermatheca^34^. Immediately following the dissections, the slides were examined using DIC optics on a Carl Zeiss 510 confocal microscope. To improve the adhesion of the sperm cells to the slide, slides were pre-treated with Poly-L-Lysine. A 4-5 μL drop of 100% Poly-L-Lysine was placed at the center of the charged surface of a ColorFrost Plus slide. A second slide was placed on top of it in such a way that both charged surfaces were facing each other and a thin film of liquid formed between them. Once the film was formed, slides were separated by sliding them over each other. Subsequently, slides were baked at 60 °C for 1–2 h.

To identify the sperm, which were often very small, we added Hoechst 33342 (Sigma-Aldrich) to the sperm media at a final concentration of 100 μg/mL. Hoechst helped to visualize and identify the characteristic condensed shape of the sperm nucleus. Sperm size was measured as the cross sectional area of the spermatid using the Carl Zeiss confocal software. 61 male sperm and 67 hermaphrodite sperm were measured. Because the group sizes to be compared were unequal, we used the Welch’s *t*-test to test if there are differences in the sperm size mean between of hermaphrodites and males^38^.

### Soporific behavior

In nematodes, males attempting to mate may induce soporific behavior, which results in the reduction of locomotory behavior of the opposite sex^39^. Soporific behavior was quantified by placing 4–5 males in a twelve-well plate seeded with bacteria with either one hermaphrodite or female. Time was recorded for how long the hermaphrodite or female stopped moving as the male started mating. 21 females and 15 hermaphrodites were tested in total.

### Chemotaxis assays

**Figure 3:**
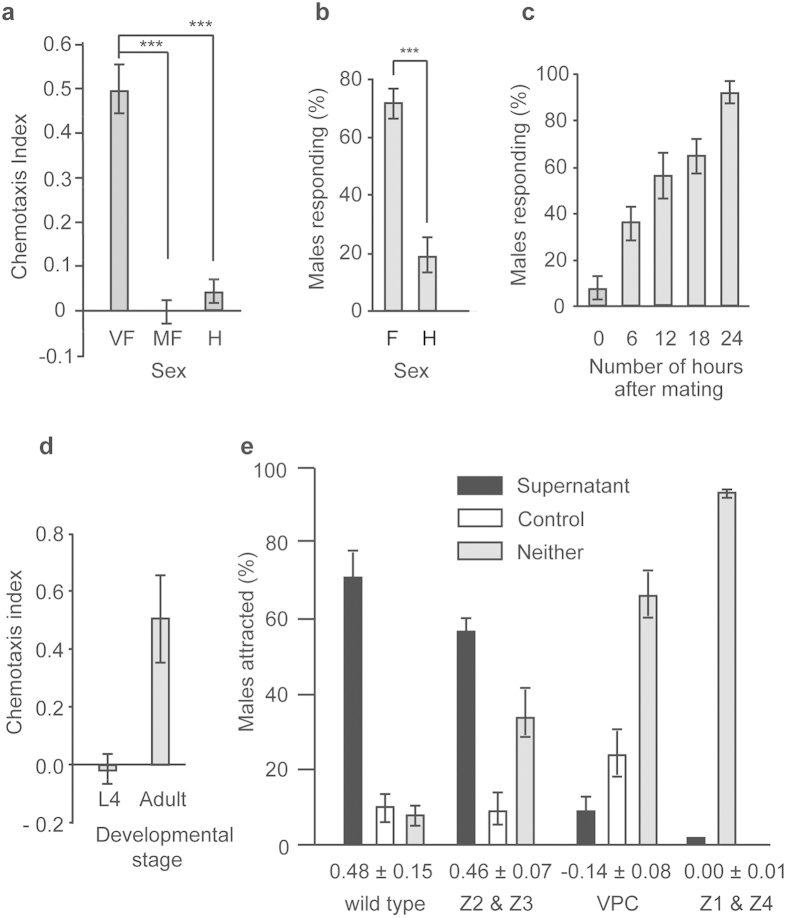
Attraction of SB347 females and males to the opposite sex. (**a**) Virgin females (VF), but not mated females (MF) or hermaphrodites (H), are attracted to supernatant of males (one-way ANOVA, ***P < 0.001). (**b**) The percentage of males that attempt to mate is higher when they are exposed to females (F) than to hermaphrodites (H) (*t*-test(6) = 6.1, ***P < 0.001). (**c**) The percentage of males that attempt to mate to females increases over time after mating. (**d**) Chemotaxis index (CI) of males to females in the fourth larval stage (L4) or as adults (see Materials and Methods for how to calculate CI). (**e**) Percentage of males attracted to unablated animals (wild type), and ablated animals for precursors of germline (Z2 & Z3), vulva precursor cells (VPC) and precursors of somatic gonad (Z1 & Z4). The CI value ± SEM is below the graph. The graphs in (**a**,**b–e**) represent the mean ± SEM of ten independent experiments with 15 males each. The graphs in (**b**,**c**) represent the mean ± SEM of five independent experiments.

To quantify the degree of attraction of worms to conditioned medium produced by different sexes, we used chemotaxis assays. Conditioned medium (henceforth referred to as ‘supernatant’) was generated by placing 5–10 worms in 100 μL of M9 buffer for 12 h at room temperature. The supernatant was stored in 10 μL aliquots at −20 °C until required. The assay was performed either on a microscope slide with a layer of 1.5% agar placed on top of an empty plate, or on a 6 cm NGM plate. 3 μL of supernatant was added on one side of the slide and 3 cm apart from the control drop that just contained M9. 15 young-adults were placed on the midline between the spots. After 30 min, worms were scored on the basis of their location. The values for the chemotaxis index (CI) shown in Fig. 3a,d,e (in x axis) and S1 were calculated with at least ten replicates for each condition. To calculate the CI, the number of worms attracted to the test spot were subtracted by the number of worms in the control spot, and divided by the total number of males assayed^40^. A CI of one indicates that all worms are attracted to the chemical in the test spot, whereas a CI close to zero indicates no attraction.

For Fig. 3a,c, ‘mated females’ were generated by placing single virgin females in contact with 4–5 males for 6 h. For Fig. 3c, 25 mated females were divided in 5 sets. 15 males were added at 5 time points (0, 6, 12, 18 and 24 h) after the initial mating to each set. The scoring and conditions of the plate were performed in the same manner as described above.

### Male response efficiency assay

The male response efficiency assay measures the percentage of males that attempt to mate with the opposite sex^41^. One day before the experiment, NGM plates were spotted with 13 μL of *E. coli* OP50-1 culture. Before the experiment, 5 males were placed for 10 min in the center of the ~5 mm diameter bacterial spot to let them acclimatize. 15 animals of the opposite sex (hermaphrodites or females) were added to the bacterial spot and observed for 4 min. If a male attempted to mate, as determined by the male tail scanning the body of the other sex, the male response was scored with the number 1. In case there was no attempt, the response was scored as 0. The behavior of each male was recorded only once. For each type of mating (male/hermaphrodite or male/female), 5 replicates were performed. To determine if the mean percentage of males attempting to mate was different when encountering a hermaphrodite or a female, we performed a two sample Student’s *t-*test (Fig. 3b).

### Laser ablation of gonadal and vulval precursor cells of female and hermaphrodite animals

To determine the site of secretion of the sex pheromone, we made a series of ablations of candidate tissues. Gonadal (Z1–Z4) and vulva precursor cells (P5.p, P6.p and P7.p) of mid- L1 animals (females or hermaphrodites) were ablated using laser microsurgery. For ablation, worms were placed on agar pads on microscopic slides. Worms were recovered after ablation using a drop of M9 buffer and transferred to a normal seeded plate. Two days after recovery, laser ablated adult worms were used to collect supernatant for attraction assays (Fig. 3e). The collection of the supernatant, and the male attraction assays were performed as described above in ‘Chemotaxis assays’.

### Exo-metabolome sample preparation

To determine the chemical nature of the sex pheromone, we generated large amounts of conditioned media for chemical analysis. 2 L of a three-week mixed stage culture of SB347 in S-complete media with 2% w/v OP50 at 20 °C was centrifuged at 13000 g followed by filtration to remove bacteria. The supernatant collected was frozen over a dry ice-acetone bath, lyophilized to a fine powder, and extracted with 100 mL of a 95:5 mixture of ethanol and water for 16 h. The resulting exo-metabolome sample was concentrated *in vacuo*, resuspended in methanol, filtered, and used for HPLC-MS/MS without further processing. Single-sex exo-metabolome samples were obtained from 800 virgin females, males, and hermaphrodites by incubating the individuals in 800 μL M9 buffer for 18 h. After centrifugation, the culture supernatants were lyophilized as described above and extracted with 1 mL of methanol each. The extracts were concentrated *in vacuo*, resuspended in methanol, filtered, and used for selective ion monitoring (SIM)-HPLC-MS analysis.

### HPLC-MS/MS, and SIM-HPLC-MS protocols

High-performance liquid chromatography with tandem mass spectrometry (HPLC-MS/MS) and selective ion monitoring (SIM)-HPLC-MS were performed using an Agilent 1100 Series HPLC system equipped with an Agilent Eclipse XDB-C18 column (9.4 × 250 mm, 5 μm particle diameter) connected to a Quattro II spectrometer (Micromass/Waters) using a 10:1 split. For HPLC, a 0.1% acetic acid-acetonitrile solvent gradient was used at a flow rate of 3.6 mL/min, starting with an acetonitrile content of 5% for 5 min which was increased to 100% over a period of 40 min. Exo-metabolome samples were analyzed by HPLC-ESI-MS in negative ion mode using a capillary voltage of 4.0 kV and a cone voltage of −40 V. HPLC-MS/MS screening for precursor ions of m/z = 73.0 performed using argon as collision gas at 2.1 mtorr and 40 eV. For the analysis of exo-metabolome samples of mixed-stage SB347 liquid cultures, the mass spectrometer was operated in scanning mode for a mass range of *m/z* 200–700. For exo-metabolome samples of single-sex worm cohorts, the spectrometer was operated in selective ion monitoring (SIM) mode and the following ions were selectively observed: *m/z* = 247 (ascr#9) and *m/z* = 275 (ascr#1).

### Quantification of pheromones

**Figure 4:**
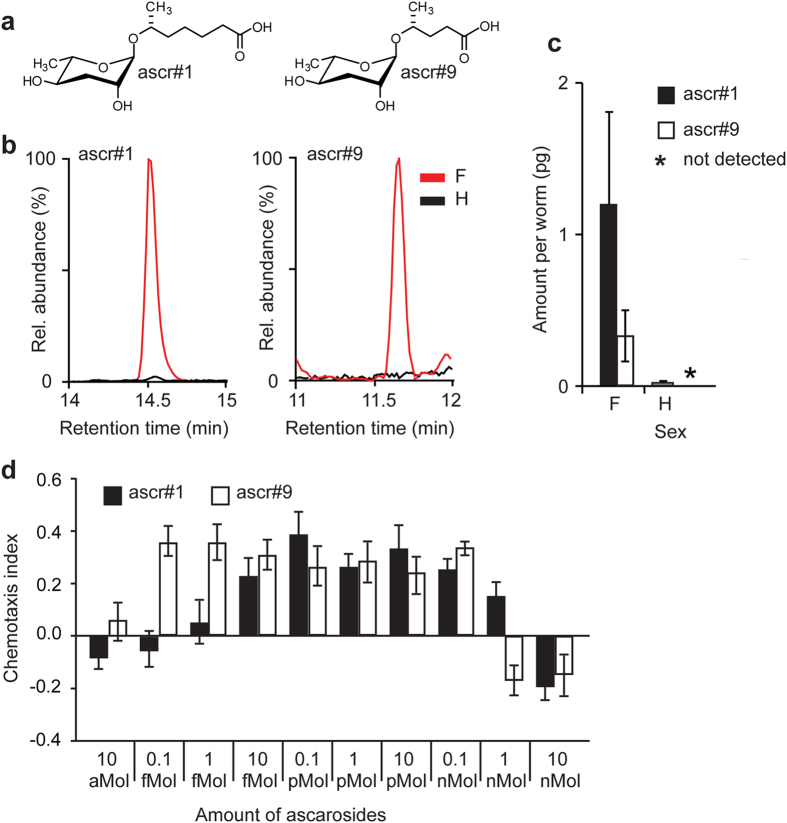
Identification of the male-attraction pheromone in *Rhabditis sp.* SB347. (**a**) Structures of ascr#1 and ascr#9. (**b**) SIM-HPLC-MS analyses showing molecular ion traces for ascr#1 (left) and ascr#9 (right) in exo-metabolome samples of females (F) and hermaphrodites (H). (**c**) Amount of ascarosides produced per worm of each sex. (**d**) Chemotaxis index of males towards synthetic ascr#1 and ascr#9. This graph represents the mean ± SEM of ten independent experiments with 15 males each.

Quantification of ascr#1 and ascr#9 in SB347 single-sex worm cohort samples was based on integration of the SIM-HPLC-MS signals from the corresponding ion-traces (Fig. 4b). Absolute amounts of ascarosides in each sample were calculated using response factors determined with synthetic standards of ascr#1 and ascr#9. The amounts calculated were then normalized to the number of worms used for exo-metabolome preparation (n = 800) to reflect the amount of ascarosides produced by a single worm. Finally, physiological concentrations of ascr#1 and ascr#9 in samples used for male attraction assays were calculated from the ascaroside amounts per worm, taking into account the number of worms used for the assays (n = 5) and the volume of M9 (100 μL) used for incubating the single sex worm cohorts over 16 h (Fig. 4c). These experiments were performed in triplicates.

### Data management and statistics treatments

Data collection and documentation was managed with the assistance of Labguru, a laboratory management software. Means, standard error of the mean (SEM), and *P* values for Student’s two-tailed *t*-test and Welch *t*-test were performed using R. One-way ANOVA with multiple comparisons *post-hoc* testing were performed using SigmaPlot statistical software.

## Results

### Female F1s are produced mostly from young hermaphrodite and female mothers

It was previously reported that hermaphrodite mothers tend to produce more female progeny in the beginning of their adult lives^32^. To test if this was the case for female mothers as well, we sexed entire broods of crosses between male and female parents. Similar to the pattern observed for a hermaphrodite parent, in which there is a higher production of female in the first day of the mother’s adult life (Type II Wald: χ^2^_1_= 353.3, *P* < 0.001) (Fig. 2a), outcrossing also results in the production of more female progeny in the first day (Type II Wald: χ^2^_1_= 381.7, *P* < 0.001) (Fig. 2b) than in the second day.

Unfortunately we could not assess the sex ratios of crosses between hermaphrodites and males for technical reasons (see Materials and Methods). Sperm size dimorphism between hermaphrodites and males, however, suggest that they can cross. A larger male sperm correlates with a higher efficiency in outcompeting the smaller hermaphrodite sperm in some nematode species^42^. Consistent with this, the area of a SB347 male sperm is almost 5 times larger than the sperm of a hermaphrodite (9.4 μm^2^ ± 0.2 SEM vs. 1.8 μm^2^ ± 0.1 SEM). Thus, we predict that males can fertilize hermaphrodites despite the presence of hermaphrodite self-sperm.

In some trioecious nematode species, such as *Rhabdias,* there is a large discrepancy in brood sizes between a female and a hermaphrodite. A *Rhabdias* female usually produces very few progeny (1–4 F1s), whereas a hermaphrodite produce thousands of F1s^26^,^43^,^44^,^45^. In SB347, however, the difference in progeny number between a female (296 ± 20 SEM F1s) and a selfing hermaphrodite mother (377 ± 21 SEM F1s) is minimal.

The mean percentage of male progeny produced over the entire reproductive period is usually below 5% for both selfing and outcrossing parents (Table 1). The percentage of male progeny that we observed for selfing hermaphrodite parents is lower than previously reported^31^. However, variations in the degree of inbreeding of the strain, sample size and the methodology used (we counted entire broods and controlled for potential escaping worms) could explain the difference.

### Mutual sexual attraction between SB347 males and females

The presence of females in trioecious species is puzzling because theory predicts that selfing hermaphrodites will rapidly outcompete them^11^,^46^. To understand the stability of the trioecious system, we aimed to characterize differences in mating behavior between hermaphrodites and females.

We observed that upon introduction of the male spicules into the vulva, SB347 females stop locomotion for a longer period than hermaphrodites (35 ± 3 sec SEM for females, 10 ± 1 sec for hermaphrodites, *t*-test(22.9)= 8.1*, P* < 0.001). This soporific behavior is more difficult to observe when males attempt to mate with hermaphrodites, because they constantly move and seem to avoid males.

In some obligatory outcrossing nematodes, females sense chemical signals produced by males^47^. To test if this was the case for SB347, we produced conditioned medium obtained from males. In contrast to hermaphrodites, females are attracted to male conditioned medium (Fig. 3a). However, once females mate with males, they lose the attraction to them.

To determine the mating preferences of the male, we used an assay that measures the proportion of males that attempt to copulate with the opposite sex within a given period^41^. We observed that more males attempt to mate with females than with hermaphrodites (Fig. 3b). The preference of male response towards females could be due to a signal that is subject to natural selection (e.g., pheromone) or a cue that is not (e.g., a waste product)^48^. To distinguish between these alternatives, we tested whether the female response to males, and the secretion of the chemical, is dependent on life stage and mating status^49^. We found that males are not attracted to females in the first hour after mating, and that females recover their attractiveness after about 24 h (Fig. 3c). Furthermore, we found that the putative sex pheromone is secreted only during adulthood, and not during larval stages (Fig. 3d). This suggests that the secretion of the male attractant is regulated, and is therefore consistent with the characteristics of a sex-pheromone that is subject to natural selection. In *C. elegans*, some pheromones are produced in the gut and later released into the medium by unknown mechanisms^50^. To determine the site of secretion of the pheromone, we suppressed vulva development by ablating the vulva precursor cells (VPCs) or the precursors of the somatic gonad (Z1 and Z4)^31^. Females lacking a vulva do not attract males, indicating that the pheromones are secreted through this organ (Fig. 3e). The ablation of the germline (Z2 and Z3), which does not affect vulva development, results in female adults that can still attract males.

### Ascarosides produced by SB347 females attract males

To elucidate the chemical structure(s) of the sex pheromone, we analyzed exo-metabolome samples of SB347 females and hermaphrodites for the presence of any of the ~170 ascarosides recently identified from *C. elegans* and other nematodes^47^,^51^,^52^, using a combination of HPLC-MS/MS and highly sensitive selective ion monitoring (SIM)-HPLC-MS. Ascarosides are nematode-specific small molecules that serve a wide variety of signaling functions that include the regulation of mating and larval development^52^,^53^,^54^. We detected two ascarosides, ascr#1 and ascr#9 (Fig. 4a)^53^ in the exo-metabolome samples of females, whereas no significant amounts of ascarosides were detected in exo-metabolome samples of hermaphrodites (Fig. 4b). To determine if the concentration of ascarosides found to be attractive to males is physiological, we measured the amount of ascarosides produced per worm (see Materials and Methods). As shown in Fig. 4c, individual females produce ascr#1 and ascr#9 in the picogram range, which is consistent with physiological levels. By testing synthetic ascr#1 and ascr#9 in the male attraction assay, we found that males are attracted to both compounds at femtomolar amount (Fig. 4d). At high nanomolar amounts, however, they are repulsive to males (Fig. 4d).

Hermaphrodites and females are not attracted to ascr#1 and ascr#9 at concentrations that show robust male attraction (Fig. S1), indicating that these ascarosides are sex-specific attractants, consistent with a function as mating signals.

### Data

Data files are deposited in http://dx.doi.org/10.6084/m9.figshare.1599794

## Discussion

In this study we found that SB347 males are more likely to cross with females than with hermaphrodites. Females produce potent chemicals, ascarosides ascr#1 and ascr#9, that attract males. These small-molecule signals are only produced by adults, can be detected from a distance, and their effect ceases for a few hours after mating. Such characteristics suggest that they are likely to be sex pheromones, shaped by natural selection, and not simply chemical cues. We note, however, that our experiments do not exclude the possibility that males are also attracted to hermaphrodites when tested with more sensitive assays^54^,^55^.

We found that for both selfing and outcrossing, SB347 female progeny is mostly produced in the first day of the mother’s life. As the probability of death becomes progressively higher with the age of the mother, the production of SB347 females by young mothers assures outcrossing and thus recombination. The mechanism underlying the production of females is not known, but it is possible that young mothers relay a female-promoting factor to early progeny.

Currently we are unable to conclude if there is selection for the maintenance of males and females in *Rhabditis* sp. SB347, as our studies are limited to one strain. Analysis of more strains exhibiting variability in the production of each sex will clarify the selection pressure for outcrossing in this species. It could be speculated that there is selection for selfing in SB347, because males are produced in low proportions. Furthermore, hermaphrodites seem to reproduce mainly by self-fertilization. In specific ecological contexts, however, the smaller frequency of males is adaptive because it minimizes competition between brothers^56^. On the other hand, selection for males and females may be favored when outcrossed offspring has a higher fitness than selfed offspring^57^,^58^. More data on the ecology of this species is necessary to address the importance of outcrossing.

The persistence of females in SB347 may also be explained if the production of females and hermaphrodites is non-overlapping in their natural habitat, as found in trioecious nematodes of the family Rhabdiasidae. In these nematodes, each generation has a different mode of reproduction^26^,^27^,^28^ and consequently hermaphrodites and females do not occur simultaneously. The parasitic generation, which is composed solely of XX selfing hermaphrodites, produces XO male and XX female progeny that breed outside of the host^59^. Male and female parents generate offspring composed of infective larvae, which become adult upon invading the host. Interestingly, SB347 is similar to Rhabdiasidae and other parasitic nematode species because crosses between SB347 females and males result in a high percentage (90%) of dauers, which later develop into self-propagating adults^60^. Another similarity is that SB347 generates extremely few (~ 3%) F1 males^34^.

The constant production of stress-resistant dauers by SB347 hermaphrodites and females is consistent with a ‘boom and bust’ type of lifestyle. This is characterized by a rapid population growth followed by a sudden crash due to the depletion of resources. The continuous dauer production could act as a protective measure against sudden changes in the environment. A single migratory SB347 dauer is specified to develop into a self-fertilizing adult, and could therefore colonize a new environment even without a mating partner. Further studies on the ecology of SB347 will clarify the significance of this developmental link. So far, only two natural isolates are known, one from a deer tick that was used as nematode bait in Rhode Island (USA)^31^ and the other from a dead beetle in West Virginia (USA) (T. Grana, personal communication, May 2015). It is unclear whether these associations are specific, or if they are of necromenic or phoretic in nature.

A simple mechanistic model for the production of three sexes is based on different genotypes for each sex. *C. elegans*, a hermaphrodite/male species, can be artificially converted to a trioecious system by introducing a mutant with a recessive allele that prevents sperm production in hermaphrodites (e.g., *fog-2* or *spe-27*)^11^,^46^. Thus, homozygous mutant XX animals are phenotypic ‘females’, XX heterozygous or wild type animals are hermaphrodites and XO animals of any genotype are males. We do not know if SB347 is a trioecious species derived from an androdioecious ancestor, or from a gonochoristic ancestor. Phylogenetic studies using other closely related species will clarify this question.

Since fertilized females and hermaphrodites in SB347 are morphologically identical, it is very difficult to distinguish them. It is therefore possible that many other nematode species with three sexes exist^29^,^61^ and that they are more prevalent than previously anticipated. Only with careful analysis, by growing larvae in isolation until adulthood, is it possible to ascertain the presence of females when hermaphrodites and males are present in culture. Further research will be necessary to determine the ecological factors that favor the co-occurrence of three sexes as an evolutionarily stable strategy.

## Additional Information

**How to cite this article**: Chaudhuri, J. *et al.* Mating dynamics in a nematode with three sexes and its evolutionary implications. *Sci. Rep.* **5**, 17676; doi: 10.1038/srep17676 (2015).

## Supplementary Material

Supplementary Figure

## Figures and Tables

**Figure 1:**
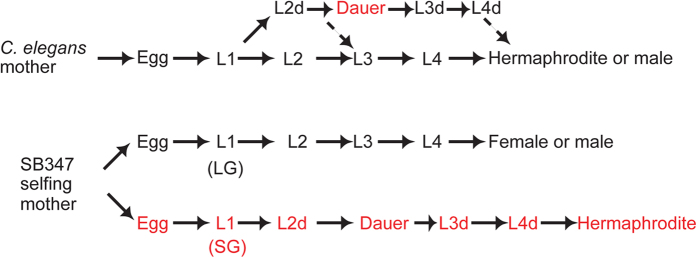
Development of *C. elegans* and SB347. In *C. elegans*, the first larval stage (L1) can facultatively develop into a stress-resistant dauer stage when exposed to unfavorable growth conditions. The initial progeny of SB347 selfing hermaphrodites is largely composed of L1s with a large gonad (LG) primordium. These larvae develop into males or females. Later progeny have a smaller gonad (SG) that obligatorily undergo dauer formation independently of growth conditions, later becoming hermaphrodites.

**Table 1:** Sex ratios of SB347 progeny of whole broods from selfing and crossing parents.

Parents	% F1 Hermaphrodite	% F1 Female	% F1 male	Total number of F1
Selfing hermaphrodite	75.7	20.7	3.6	7543
Female x male	91.1	6.0	2.9	5642

## References

[b1] Weeks S. C. The role of androdioecy and gynodioecy in mediating evolutionary transitions between dioecy and hermaphroditism in the Animalia. Evolution 66, 3670–3686 (2012). 23206127 10.1111/j.1558-5646.2012.01714.x

[b2] Pannell J. R. The evolution and maintenance of androdioecy. Annu Rev Ecol Syst 33, 397–425 (2002).

[b3] Darwin C. The effects of cross and self fertilisation in the vegetable kingdom. (D. Appleton and company, 1877).

[b4] Charlesworth D. Androdioecy and the evolution of dioecy. Biol J Linn Soc 22, 333–348 (1984).

[b5] Perry L. E., Pannell J. R. & Dorken M. E. Two’s company, three’s a crowd: experimental evaluation of the evolutionary maintenance of trioecy in *Mercurialis annua* (Euphorbiaceae). PLoS One 7, e35597 (2012). 22532862 10.1371/journal.pone.0035597 PMC3330815

[b6] Renner S. S. & Ricklefs R. E. Dioecy and its correlates in the flowering plants. Am J Bot 82, 596–606 (1995).

[b7] Charlesworth D. & Wright S. I. Breeding systems and genome evolution. Curr Opin Genet Dev 11, 685–690 (2001). 11682314 10.1016/s0959-437x(00)00254-9

[b8] Ghiselin M. T. The evolution of hermaphroditism among animals. Q Rev Biol 44, 189–208 (1969). 4901396 10.1086/406066

[b9] Eppley S. M. & Jesson L. K. Moving to mate: the evolution of separate and combined sexes in multicellular organisms. J Evol Biol 21, 727–736 (2008). 18373660 10.1111/j.1420-9101.2008.01524.x

[b10] Chasnov J. R. The evolution from females to hermaphrodites results in a sexual conflict over mating in androdioecious nematode worms and clam shrimp. J Evol Biol 23, 539–556 (2010). 20074309 10.1111/j.1420-9101.2009.01919.x

[b11] Stewart A. D. & Phillips P. C. Selection and maintenance of androdioecy in *Caenorhabditis elegans*. Genetics 160, 975–982 (2002). 11901115 10.1093/genetics/160.3.975 PMC1462032

[b12] Hedgecock E. M. The mating system of *Caenorhabditis elegans*, evolutionary equilibrium between self- and cross-fertilization in a facultative hermaphrodite Amer Nat 110, 1007–1012 (1976).

[b13] Chasnov J. R. & Chow K. L. Why are there males in the hermaphroditic species *Caenorhabditis elegans*? Genetics 160, 983–994 (2002). 11901116 10.1093/genetics/160.3.983 PMC1462001

[b14] Theologidis I., Chelo I. M., Goy C. & Teotonio H. Reproductive assurance drives transitions to self-fertilization in experimental *Caenorhabditis elegans*. BMC Biol 12, 93 (2014). 25369737 10.1186/s12915-014-0093-1 PMC4234830

[b15] Wolf D. E. & Takebayashi N. Pollen limitation and the evolution of androdioecy from dioecy. Amer Nat 163, 122–137 (2004). 10.1086/380493 14767842

[b16] Kaliszewicz A. Interference of asexual and sexual reproduction in the green hydra. Ecol Res 26, 147–152 (2011).

[b17] Armoza-Zvuloni R., Kramarsky-Winter E., Loya Y., Schlesinger A. & Rosenfeld H. Trioecy, a unique breeding strategy in the sea anemone *Aiptasia diaphana* and its association with sex steroids. Biol Reprod 90, 122 (2014). 24790160 10.1095/biolreprod.113.114116

[b18] Mayer W. E., Herrmann M. & Sommer R. J. Phylogeny of the nematode genus *Pristionchus* and implications for biodiversity, biogeography and the evolution of hermaphroditism. BMC Evol Biol 7, 104 (2007). 17605767 10.1186/1471-2148-7-104 PMC1929057

[b19] Shinya R., Hasegawa K., Chen A., Kanzaki N. & Sternberg P. W. Evidence of hermaphroditism and sex ratio distortion in the fungal feeding nematode *Bursaphelenchus okinawaensis*. G3 (Bethesda) 4, 1907–1917 (2014). 25122669 10.1534/g3.114.012385 PMC4199697

[b20] Mayer W. E., Herrmann M. & Sommer R. J. Molecular phylogeny of beetle associated diplogastrid nematodes suggests host switching rather than nematode-beetle coevolution. BMC Evol Biol 9, 212 (2009). 19703296 10.1186/1471-2148-9-212 PMC2737313

[b21] Kiontke K. *et al.* *Caenorhabditis* phylogeny predicts convergence of hermaphroditism and extensive intron loss. PNAS 101, 9003–9008 (2004). 15184656 10.1073/pnas.0403094101 PMC428462

[b22] Pires-daSilva A. Evolution of the control of sexual identity in nematodes. Semin Cell Dev Biol 18, 362–370 (2007). 17306573 10.1016/j.semcdb.2006.11.014

[b23] Kanzaki N., Ragsdale E. J., Herrmann M., Susoy V. & Sommer R. J. Two androdioecious and one dioecious new species of *Pristionchus* (Nematoda: Diplogastridae): new reference points for the evolution of reproductive mode. J Nematol 45, 172–194 (2013). 24115783 PMC3792836

[b24] Kiontke K. C. *et al.* A phylogeny and molecular barcodes for *Caenorhabditis*, with numerous new species from rotting fruits. BMC Evol Biol 11, 339 (2011). 22103856 10.1186/1471-2148-11-339 PMC3277298

[b25] Blaxter M. L. *et al.* A molecular evolutionary framework for the phylum Nematoda. Nature 392, 71–75 (1998). 9510248 10.1038/32160

[b26] Tkach V. V., Kuzmin Y. & Snyder S. D. Molecular insight into systematics, host associations, life cycles and geographic distribution of the nematode family Rhabdiasidae. Int J Parasitol 44, 273–284 (2014). 24560917 10.1016/j.ijpara.2013.12.005

[b27] Langford G. J. & Janovy J. Jr. Comparative life cycles and life histories of North American *Rhabdias* spp. (Nematoda: Rhabdiasidae): lungworms from snakes and anurans. J Parasitol 95, 1145–1155 (2009). 19348516 10.1645/GE-2044.1

[b28] Langford G. J. & Janovy J. Jr. Host specificity of North American *Rhabdias* spp. (Nematoda: Rhabdiasidae): combining field data and experimental infections with a molecular phylogeny. J Parasitol 99, 277–286 (2013). 22988815 10.1645/GE-3217.1

[b29] Sudhaus W. Phylogenetic systematisation and catalogue of paraphyletic “Rhabditidae” (Secernentea, Nematoda). J Nematode Morphol Sys 14, 113–178 (2011).

[b30] Stock S. P. Diversity, Biology and Evolutionary Relationships. In Nematode Pathogenesis of Insects and Other Pests - Ecology and Applied Technologies for Sustainable Plant and Crop Protection Vol. 1 Sustainability in Plant and Crop Protection (ed R. Campos-Herrera) Ch. 1, 3–28 (Springer Science+Business Media 2015).

[b31] Félix M. A. Alternative morphs and plasticity of vulval development in a rhabditid nematode species. Dev Genes Evol 214, 55–63 (2004). 14730447 10.1007/s00427-003-0376-y

[b32] Chaudhuri J., Kache V. & Pires-daSilva A. Regulation of sexual plasticity in a nematode that produces males, females, and hermaphrodites. Curr Biol 21, 1548–1551 (2011). 21906947 10.1016/j.cub.2011.08.009

[b33] Kiontke K. *et al.* Trends, stasis, and drift in the evolution of nematode vulva development. Curr Biol 17, 1925–1937 (2007). 18024125 10.1016/j.cub.2007.10.061

[b34] Shakes D. C., Neva B. J., Huynh H., Chaudhuri J. & Pires-daSilva A. Asymmetric spermatocyte division as a mechanism for controlling sex ratios. Nat Commun 2, 157 (2011). 21245838 10.1038/ncomms1160 PMC5885250

[b35] Stiernagle T. Maintenance of *C. elegans*. WormBook, 1–11 (2006). 18050451 10.1895/wormbook.1.101.1 PMC4781397

[b36] Avery L. The genetics of feeding in *Caenorhabditis elegans*. Genetics 133, 897–917 (1993). 8462849 10.1093/genetics/133.4.897 PMC1205408

[b37] Fox J. & Weisberg S. An R companion to applied regression. 2nd edn, (SAGE Publications, 2011).

[b38] Zimmerman D. W. A note on preliminary tests of equality of variances. Br J Math Stat Psychol 57, 173–181 (2004). 15171807 10.1348/000711004849222

[b39] Garcia L. R., LeBoeuf B. & Koo P. Diversity in mating behavior of hermaphroditic and male-female *Caenorhabditis* nematodes. Genetics 175, 1761–1771 (2007). 17277358 10.1534/genetics.106.068304 PMC1855125

[b40] Bargmann C. I. & Horvitz H. R. Control of larval development by chemosensory neurons in *Caenorhabditis elegans*. Science 251, 1243–1246 (1991). 2006412 10.1126/science.2006412

[b41] Morsci N. S., Haas L. A. & Barr M. M. Sperm status regulates sexual attraction in *Caenorhabditis elegans*. Genetics 189, 1341–1346 (2011). 21968192 10.1534/genetics.111.133603 PMC3241412

[b42] LaMunyon C. W. & Ward S. Larger sperm outcompete smaller sperm in the nematode *Caenorhabditis elegans*. Proc Biol Sci 265, 1997–2002 (1998). 9821364 10.1098/rspb.1998.0531 PMC1689481

[b43] Goodey T. The anatomy and life-history of the nematode *Rhabdias fuscovenosa* (Railliet) from the grass snake *Tropidonotus natrix*. J Helminthol II, 51–64 (1924).

[b44] Kuzmin Y. Morphology of parasitic and free-living adults of *Rhabdias rubrovenosa* (Nematoda, Rhabdiasidae). Vestn Zool 34, 109–114 (2000).

[b45] Baker M. R. Free-living and parasitic development of *Rhabdias* spp (Nematoda, Rhabdiasidae) in amphibians. Can J Zool 57, 161–178 (1979).

[b46] Cutter A. D. Mutation and the experimental evolution of outcrossing in *Caenorhabditis elegans*. J Evol Biol 18, 27–34 (2005). 15669958 10.1111/j.1420-9101.2004.00804.x

[b47] Choe A. *et al.* Sex-specific mating pheromones in the nematode *Panagrellus redivivus*. PNAS 109, 20949–20954 (2012). 23213209 10.1073/pnas.1218302109 PMC3529029

[b48] Viney M. E. & Franks N. R. Is dauer pheromone of *Caenorhabditis elegans* really a pheromone? Naturwissenschaften 91, 123–124 (2004). 15034661 10.1007/s00114-004-0503-2

[b49] Chasnov J. R., So W. K., Chan C. M. & Chow K. L. The species, sex, and stage specificity of a *Caenorhabditis* sex pheromone. PNAS 104, 6730–6735 (2007). 17416682 10.1073/pnas.0608050104 PMC1871854

[b50] Butcher R. A. *et al.* Biosynthesis of the Caenorhabditis elegans dauer pheromone. PNAS 106, 1875–1879 (2009). 19174521 10.1073/pnas.0810338106 PMC2631283

[b51] Bose N. *et al.* Complex small-molecule architectures regulate phenotypic plasticity in a nematode. Angew Chem 51, 12438–12443 (2012). 23161728 10.1002/anie.201206797 PMC3733369

[b52] von Reuss S. H. *et al.* Comparative metabolomics reveals biogenesis of ascarosides, a modular library of small-molecule signals in *C. elegans*. J Am Chem Soc 134, 1817–1824 (2012). 22239548 10.1021/ja210202y PMC3269134

[b53] Choe A. *et al.* Ascaroside signaling is widely conserved among nematodes. Curr Biol 22, 772–780 (2012). 22503501 10.1016/j.cub.2012.03.024 PMC3360977

[b54] Srinivasan J. *et al.* A blend of small molecules regulates both mating and development in *Caenorhabditis elegans*. Nature 454, 1115–1118 (2008). 18650807 10.1038/nature07168 PMC2774729

[b55] Simon J. M. & Sternberg P. W. Evidence of a mate-finding cue in the hermaphrodite nematode *Caenorhabditis elegans*. PNAS 99, 1598–1603 (2002). 11818544 10.1073/pnas.032225799 PMC122236

[b56] Hamilton W. D. Extraordinary sex ratios. Science 156, 477–488 (1967). 6021675 10.1126/science.156.3774.477

[b57] Morran L. T., Parmenter M. D. & Phillips P. C. Mutation load and rapid adaptation favour outcrossing over self-fertilization. Nature 462, 350–352 (2009). 19847164 10.1038/nature08496 PMC4183137

[b58] Morran L. T., Cappy B. J., Anderson J. L. & Phillips P. C. Sexual partners for the stressed: facultative outcrossing in the self-fertilizing nematode *Caenorhabditis elegans*. Evolution 63, 1473–1482 (2009). 19210536 10.1111/j.1558-5646.2009.00652.x PMC4183189

[b59] Runey W. M., Runey G. L. & Lauter F. H. Gametogenesis and fertilization in *Rhabdias ranae* Walton 1929: I. The parasitic hermaphrodite. J Parasitol 64, 1008–1014 (1978). 570219

[b60] Wang Z. *et al.* Identification of the nuclear receptor DAF-12 as a therapeutic target in parasitic nematodes. PNAS 106, 9138–9143 (2009). 19497877 10.1073/pnas.0904064106 PMC2695123

[b61] Maupas E. Modes et formes de reproduction des nématodes. Ann Zool Exp Gen 8, 463–624 (1900).

