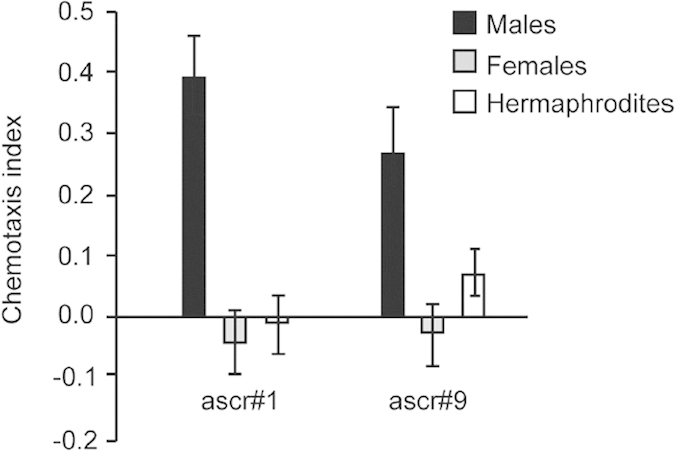# Erratum: Mating dynamics in a nematode with three sexes and its evolutionary implications

**DOI:** 10.1038/srep23852

**Published:** 2016-04-07

**Authors:** Jyotiska Chaudhuri, Neelanjan Bose, Sophie Tandonnet, Sally Adams, Giusy Zuco, Vikas Kache, Manish Parihar, Stephan H. von Reuss, Frank C. Schroeder, Andre Pires-daSilva

Scientific Reports 5: Article number: 17676; 10.1038/srep17676 Published online: 12032015; Updated: 04072016

This Article contains an error in Supplementary Figure 1 where the value of the male chemotaxis index towards ascr#9 ‘0.26 ± 0.07’ was incorrectly depicted as ‘0.38 ± 0.07’. The correct Supplementary Figure 1 appears below as Figure 1.

## Figures and Tables

**Figure 1:**