# Supplementary material for: Mating dynamics in a nematode with three sexes and its evolutionary implications
Source: Sci Rep. 2015 Dec 3;5:17676. doi: 10.1038/srep17676 (PMC4668576; doi:10.1038/srep17676)
Supplement: Supplementary Figure [file srep17676-s1.pdf]

Title: Mating dynamics in a nematode with three sexes and its evolutionary implications

Authors: Jyotiska Chaudhuri[1#], Neelanjan Bose[2#], Sophie Tandonnet[3] , Sally Adams[3], Giusy Zuco[3], Vikas Kache[1], Manish Parihar[1], Stephan H. von Reuss[2†], Frank C. Schroeder[2], Andre Pires-daSilva[1, 3]*

Affiliations:

1. University of Texas at Arlington, Department of Biology, Arlington, USA

2. Boyce Thompson Institute and Department of Chemistry and Chemical Biology, Cornell University, Ithaca, New York 14853, USA.

3. University of Warwick, School of Life Sciences, Coventry, United Kingdom.

Current Address:

# Buck Institute for Research on Aging, 8001 Redwood Blvd., Novato, California 94945, USA.

†Department of Bioorganic Chemistry, Max Planck Institute for Chemical Ecology, Jena, D-07745, Germany.

To whom correspondence should be addressed: Email: andre.pires@warwick.ac.uk (A.P.-d.S.)

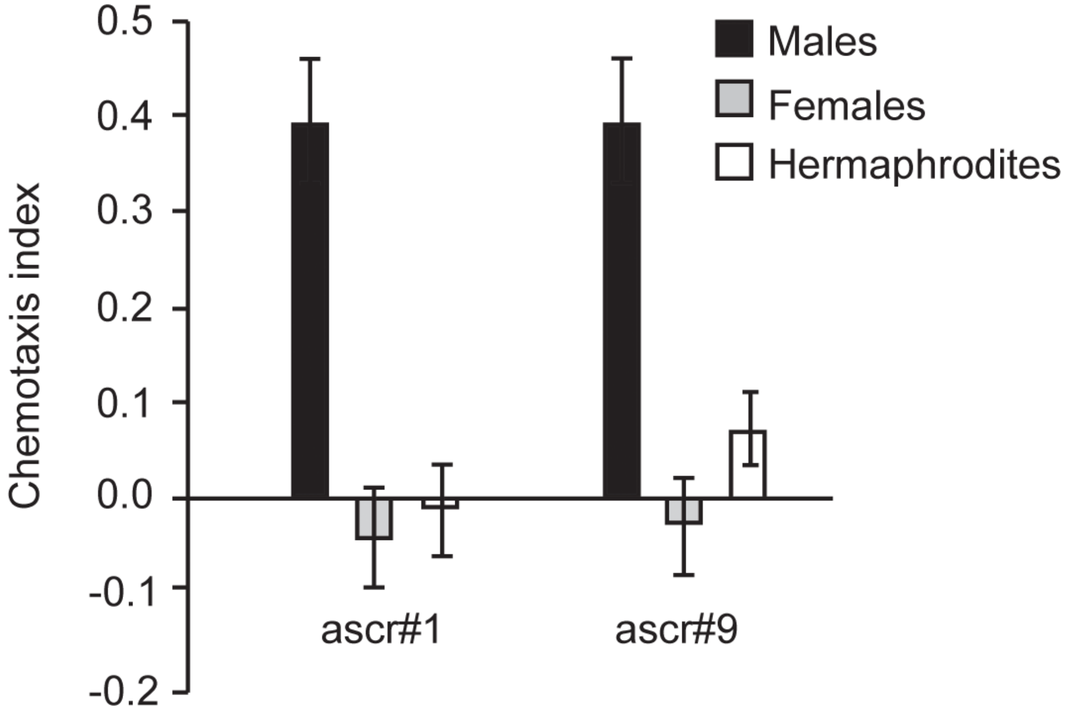

Fig. S1. Females and hermaphrodites are not attracted to ascr#1 and ascr#9. Chemotaxis index of males, females and hermaphrodites towards synthetic 0.1 pMol ascr#1 and ascr#9. The graph represents the mean ± SEM of ten independent experiments with 15 males in each.
